# Prenatal Surgery for Open Fetal Spina Bifida in Patients with Obesity: A Review of Current Evidence and Future Directions

**DOI:** 10.3390/jcm13195661

**Published:** 2024-09-24

**Authors:** Giulia Bonanni, Nikan Zargarzadeh, Eyal Krispin, Weston T. Northam, Elisa Bevilacqua, Hiba J. Mustafa, Alireza A. Shamshirsaz

**Affiliations:** 1 Maternal Fetal Care Center, Division of Fetal Medicine and Surgery, Boston Children’s Hospital, Harvard Medical School, Boston, MA 02115, USA; 2 Department of Neurosurgery, Boston Children’s Hospital, Harvard Medical School, Boston, MA 02115, USA; weston.northam@childrens.harvard.edu; 3 Department of Women, Children, and Public Health Sciences, IRCCS Agostino Gemelli University Polyclinic Foundation, Catholic University of the Sacred Heart, 00168 Rome, Italy; elisa.bevilacqua@policlinicogemelli.it; 4 Division of Maternal-Fetal Medicine, Department of Obstetrics and Gynecology, Indiana University School of Medicine, Indianapolis, IN 46202, USA; 5 Fetal Center, Riley Children’s Health, Indianapolis, IN 46202, USA

**Keywords:** fetal surgery, myelomeningocele, maternal obesity, prenatal repair, fetal spina bifida, neural tube defects

## Abstract

**Background:** Obesity rates have significantly increased globally, affecting up to 40% of women of childbearing age in the United States. While prenatal repair of open fetal spina bifida has shown improved outcomes, most fetal surgery centers exclude patients with a body mass index (BMI) ≥ 35 kg/m^2^ based on criteria from the Management of Myelomeningocele Study (MOMS) trial. This exclusion raises concerns about healthcare equity and highlights a significant knowledge gap regarding the safety and efficacy of fetal spina bifida repair in patients with obesity. **Objective:** To review the current state of knowledge regarding open fetal surgery for fetal spina bifida in patients with obesity, focusing on safety, efficacy, and clinical considerations. **Methods:** A comprehensive literature search was conducted using the PubMed and EMBASE databases, covering articles from the inception of the databases to April 2024. Studies discussing fetal surgery for neural tube defects and documenting BMI measurements and their impact on surgical outcomes, published in peer-reviewed journals, and available in English were included. Quantitative data were extracted into an Excel sheet, and data synthesis was conducted using the R programming language (version 4.3.3). **Results:** Three retrospective studies examining outcomes of prenatal open spina bifida repair in a total of 43 patients with a BMI ≥ 35 kg/m^2^ were identified. These studies did not report significant adverse maternal or fetal outcomes compared to patients with lower BMIs. Our pooled analysis revealed a perinatal mortality rate of 6.1% (95% CI: 1.76–18.92%), with 28.0% (95% CI: 14.0–48.2%) experiencing the premature rupture of membranes and 82.0% (95% CI: 29.2–98.0%) delivering preterm (<37 weeks). Membrane separation was reported in 10.3% of cases (95% CI: 3.3–27.7%), the mean gestational age at birth was 34.3 weeks (95% CI: 32.3–36.3), and the average birth weight was 2651.5 g (95% CI: 2473.7–2829.4). Additionally, 40.1% (95% CI: 23.1–60.0%) required a ventriculoperitoneal shunt. **Conclusion:** While current evidence suggests that fetal spina bifida repair may be feasible in patients with obesity, significant limitations in the existing body of research were identified. These include small sample sizes, retrospective designs, and a lack of long-term follow-up data. There is an urgent need for large-scale, prospective, multicenter studies to definitively establish the safety and efficacy of fetal spina bifida repair in patients with obesity. Such research is crucial for developing evidence-based guidelines, improving clinical outcomes, and addressing healthcare disparities in this growing patient population with obesity.

## 1. Introduction

The global prevalence of obesity has experienced a dramatic increase over the past few decades, nearly tripling since 1975 [1,2,3,4]. This trend has significant implications for public health, particularly in the context of maternal and fetal health. In the United States, up to 40% of women of childbearing age are currently affected by obesity, a statistic that underscores the urgency of addressing this issue in prenatal care and fetal interventions [1,2,3,4].

Obesity is a recognized non-genetic risk factor for fetal spina bifida. Studies have shown that patients with obesity have a 1.24 times higher likelihood of experiencing pregnancies affected by fetal spina bifida compared to those with a normal body mass index (BMI) [5,6]. This association remains significant even after controlling for multiple socioeconomic, demographic, and metabolic factors [7].

The mechanism underlying this increased risk is not fully understood, but several hypotheses have been proposed. One suggestion is that there may be a variable intake and/or a bodily distribution of folic acid in populations with obesity. Folic acid is crucial for neural tube development, and its metabolism might be altered in individuals with obesity. Additionally, abnormalities in glucose metabolism, which are more common in individuals with obesity, may contribute to the increased risk of fetal spina bifida [8,9].

The Management of Myelomeningocele Study (MOMS) trial, a landmark study in the field of fetal surgery, demonstrated that prenatal spina bifida repair significantly improves outcomes for affected children. The benefits include a reduced need for ventriculoperitoneal shunting and enhanced neurologic function to an extent that outweighs the maternal risks [10]. As a result of this groundbreaking research, open prenatal repair of fetal spina bifida has become a widely accepted therapy in fetal medicine centers around the world. However, the MOMS trial protocol excluded pregnant patients with a BMI ≥ 35 kg/m^2^ “for safety reasons”, creating a significant knowledge gap regarding the safety and efficacy of this procedure in patients with obesity. The rationale behind this exclusion was based on concerns about increased surgical risks. However, it also meant that a substantial portion of the patient population who might benefit from this intervention was not represented in the trial results.

Many fetal surgery centers continue to adhere to the MOMS eligibility criteria, potentially limiting access to this beneficial intervention for a substantial portion of the patient population [10,11,12]. This situation raises significant ethical concerns related to equity and access to medical care. The implications are particularly troubling given that obesity is more prevalent among certain racial and ethnic minorities and lower socioeconomic groups, potentially exacerbating existing health disparities.

A retrospective study found that for every unit increase in BMI, the odds of undergoing open fetal surgery for fetal spina bifida decrease by almost one unit [13]. Furthermore, a 2016 survey sent to the 17 American fetal therapy centers participating in the Fetal Myelomeningocele Consortium revealed that only 9% of participating fetal surgery centers would consider surgery for patients with BMIs up to 40 kg/m^2^, highlighting a significant disparity in care [14].

The field of fetal surgery has yet to fully adapt to the challenges posed by maternal obesity, with no established guidelines or comprehensive research addressing these issues. Understanding the safety and efficacy of prenatal interventions in patients with obesity is crucial for improving clinical outcomes addressing existing socio-demographic disparities [13,15,16], ensuring equitable access to prenatal surgery, and avoiding unjustly denying interventions based on BMI alone. This review aims to provide a comprehensive overview of the current state of knowledge regarding the safety, efficacy, and clinical considerations of open fetal surgery for fetal spina bifida in patients with obesity. By synthesizing the available data and identifying knowledge gaps, we seek to highlight the urgent need for focused research and the development of tailored guidelines for this growing patient cohort.

## 2. Materials and Methods

Our methodology was designed to capture the most relevant and up-to-date information on the topic of open fetal surgery for spina bifida repair in patients with obesity. A comprehensive literature search was conducted using the PubMed and EMBASE databases to identify relevant studies examining the impact of obesity at the time of surgery on open fetal repair for neural tube defects. The search period for these publications extended from the inception of the databases to April 2024. The search strategy incorporated terms related to fetal surgery for neural tube defects and obesity to ensure a broad retrieval of relevant articles, such as but not limited to ‘fetal surgery’, ‘spina bifida’, ‘obesity’, ‘body mass index’, and ‘maternal weight’.

Articles were included if they met all the following criteria: (i) Content: Articles were required to discuss fetal surgery for neural tube defects, including specific details about the procedures and outcomes. (ii) Obesity focus: The articles needed to address aspects related to maternal obesity, with a focus on documented BMI measurements and their impact on surgical outcomes. (iii) Publication type: Only articles published in peer-reviewed journals were included. (iv) Language: Articles were required to be available in English, ensuring that we could accurately interpret and analyze the included research. In addition to the search results, we conducted a manual review of the references from pertinent articles identified through our search strategy.

One reviewer (G.B.) performed an initial screening of titles and abstracts to exclude irrelevant studies. The remaining articles were then subjected to a full-text review to ensure they met all the inclusion criteria. Key information was extracted from eligible studies, focusing on study design and methodology, population characteristics, and outcomes related to maternal obesity. The results from the selected studies were grouped thematically to provide a comprehensive overview of the current state of knowledge.

For quantitative analysis, we extracted the data into an Excel sheet and performed data synthesis using the R programming language (version 4.3.3). Data analysis was conducted with the “meta” and “metafor” packages, which facilitated the pooling of proportions and means.

Pooled outcomes of studies on open fetal spina bifida in patients with obesity were presented alongside data from the MOMS trial. To provide context for our findings, we presented pooled outcomes of studies on open fetal spina bifida repair in patients with obesity alongside data from the MOMS trial. The MOMS trial was chosen as a comparison point for several reasons: (i) It is the foundational study that established prenatal surgery for open spina bifida as a standard of care. (ii) It offers essential outcomes for a cohort of patients with a BMI < 35, providing a crucial reference point for our analysis. (iii) Its rigorous methodology and sample size make it a reliable benchmark for comparison. This comparative approach allows us to contextualize the outcomes calculated for patients with a BMI > 35, highlighting any similarities or differences in outcomes between the two groups.

## 3. Results

### 3.1. Study Characteristics

We identified four primary studies that did not adhere to the BMI-related MOMS criteria at the time of surgery. The characteristics of these studies are summarized in Table 1, which provides an overview of the study designs, patient populations, and key outcomes measured.

The first retrospective cohort, conducted at Children’s Hospital Colorado and published in 2019 by Hilton et al., included 11 patients with a BMI between 35 and 40 kg/m^2^, with a mean BMI of 37.4 ± 1.5 kg/m^2^ [17]. The authors descriptively presented their results alongside those from the MOMS trial to highlight clinical relevance rather than to imply a formal statistical comparison. The mean gestational age at delivery was 2 weeks earlier compared to the MOMS trial (32 vs. 34 gestational weeks). No clinically significant differences in adverse effects related to obesity were observed compared to the MOMS cohort, including preterm premature rupture of membranes (pPROM), pulmonary edema, hysterotomy, uterine dehiscence status at delivery, perinatal death, or shunt placement. There was one perinatal death immediately following the fetal intervention, and the shunt rate at 1 year was 45% (5/11 patients) compared to the 40% rate reported in the MOMS trial [17]. This study provided initial low-quality evidence that fetal surgery outcomes in patients with obesity might be comparable to those in patients with lower BMIs, despite the earlier gestational age at delivery.

In 2020, Moldenhaur et al. stratified outcomes data from a retrospective cohort of 264 patients from the Children’s Hospital of Philadelphia, including 196 (74.2%) with a BMI < 30 kg/m^2^, 54 (20.5%) with a BMI of 30–34.99 kg/m^2^, and 14 (5.3%) with a BMI of 35–40 kg/m^2^ [18]. The mean BMI for the highest BMI cohort was 37.3 ± 1.4 kg/m^2^. Increasing maternal BMI did not result in a negative impact on maternal, obstetric, and perinatal outcomes. Operative time increased with increasing BMI (74.6 min vs. 78.8 min vs. 88.1 min; *p* < 0.0001); otherwise, other perioperative outcomes were similar between the groups. Notably, the rate of preterm delivery at < 37 weeks was lowest in the highest BMI group (*p* = 0.049) [18].

More recently, a retrospective study by Zepf et al. published data on 192 patients from the Zurich Center for Fetal Diagnosis and Therapy, including 146 (76.0%) with a BMI < 30 kg/m^2^, 28 (14.6%) with a BMI of 30–35 kg/m^2^, and 18 (9.4%) with a BMI ≥ 35 kg/m^2^ [20]. The median and interquartile range for BMI at surgery for the highest BMI group was 37.4 kg/m^2^ (interquartile range: 36.2–41.5), with no upper limit defined. This study did not show any clinically relevant differences in maternal and/or fetal outcomes among women with a BMI ≥ 35 kg/m^2^ undergoing open fetal spina bifida repair compared to patients with a BMI < 30 kg/m^2^ or a BMI of 30–35 kg/m^2^. The frequency of maternal wound seroma was significantly higher in women with a BMI ≥ 35 kg/m^2^ (*p* = 0.04), but this did not reflect a higher need for interventions. In contrast to Hilton et al., no significant difference in gestational age at birth was seen between the groups (*p* = 0.92). However, a complication that occurred significantly more often in patients with a BMI ≥ 35 kg/m^2^ was vaginal bleeding (*p* = 0.01), although the bleeding intensity was less than menstrual flow in all cases [20].

### 3.2. Obstetrical and Perinatal Outcomes

**Figure 1:**
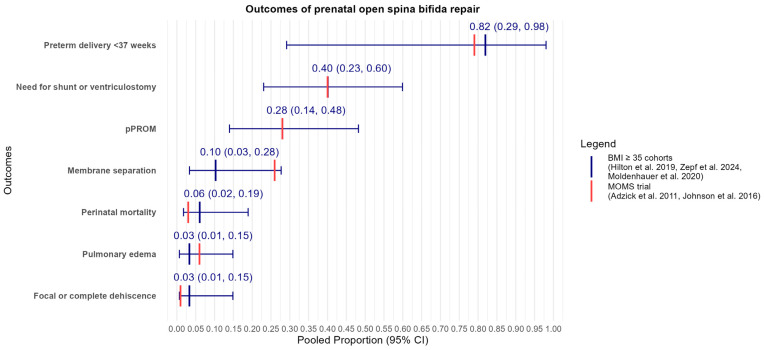
Pooled proportions of outcomes with 95% confidence intervals (CIs) in cohorts of patients with BMI ≥ 35 kg/m^2^ undergoing prenatal open spina bifida repair [17,18,20,21] (*blue*) along with MOMS Trial data for patients with BMI < 35 kg/m^2^ [10] (*red*). Each blue line represents the pooled proportion of a specific outcome, with error bars indicating the 95% confidence interval for patients with BMI ≥ 35 kg/m^2^.

To provide a comprehensive overview of the outcomes of prenatal spina bifida repair in patients with obesity, we pooled data from the available studies. Figure 1 and Table 2 present these pooled outcomes for patients with a BMI ≥ 35 kg/m^2^. Perinatal mortality was 6.1% (95% CI: 1.8–18.9%, I^2^ = 0.0%). The incidence of pPROM was 28.0% (95% CI: 14.0–48.2%, I^2^ = 0.0%), and preterm delivery at < 37 weeks was 82.0% (95% CI: 29.2–98.0%, I^2^ = 0.63%). The mean duration of surgery was 118.4 min (95% CI: 58.7–178.0, I^2^ = 0.99%), and gestational age at birth was 34.31 weeks (95% CI: 32.3–36.3, I^2^ = 0.78%). Birth weight averaged 2651.5 g (95% CI: 2473.7–2829.4, I^2^ = 0.0%). The need for a shunt was observed in 40.1% of cases (95% CI: 23.1–59.9%, I^2^ = 0.0%). Membrane separation was reported in 10.3% of cases (95% CI: 3.3–27.7%, I^2^ = 0.0%). Importantly, there were no cases of pulmonary edema and focal or complete dehiscence of the hysterotomy site at delivery in each of the three surgical groups, highlighting a key aspect of safety.

Table 2 provides a comparative overview by juxtaposing these outcomes with those from the MOMS trial, which only included patients with a BMI < 35 kg/m^2^. The similarities suggest that outcomes for higher BMI patients may be closely aligned with those for lower BMI patients.

## 4. Discussion

Recent retrospective studies including patients with BMIs of 35–40 kg/m^2^ (class II obesity) or higher—such as the study by Zepf et al., which included 6 patients with BMIs > 40 kg/m^2^ (class III obesity)—have not identified significant maternal and/or fetal outcome problems related to fetal spina bifida prenatal surgeries in patients with obesity [18,19,20]. The low perinatal mortality rate and minimal incidence of pulmonary edema we found when pooling the data from these studies suggest that prenatal open spina bifida repair is relatively safe in this high-risk group. These findings challenge the current practice of stringent BMI-related eligibility criteria and suggest that a more liberal approach toward patients with BMIs ≥ 35 kg/m^2^ may be justified, provided they do not exhibit serious body weight-associated problems that could worsen during pregnancy [19].

However, it is essential to recognize that these studies are not without limitations. All three investigations were retrospective and had relatively small sample sizes, particularly for patients with a BMI ≥ 35 kg/m^2^, and lacked pre-pregnancy BMI data for all patients. The single-institution design of the studies, along with the challenges in tracking long-term outcomes due to referral patterns, may not have captured all potential complications, especially those occurring after discharge. These factors limit the generalizability of the findings and underscore the need for caution when extrapolating these results to broader populations. Additionally, it is important to note that the data discussed focus primarily on open fetal spina bifida repair. Emerging techniques, such as laparotomy-assisted fetoscopy, are increasingly being explored as less invasive alternatives. However, these newer techniques have even more limited data and may present additional technical challenges, such as visualization difficulties and instrument maneuverability, especially in pregnancies with obesity.

These limitations highlight the need for large-scale, multicenter prospective studies to more definitively assess the safety and efficacy of fetal spina bifida repair in pregnancies with obesity. Such research is crucial for providing more robust evidence that can guide clinical decision making and potentially expand access to this vital intervention for a broader range of patients. An important initiative supported by the North American Fetal Therapy Network (NAFTNet, https://www.chop.edu/centers-programs/center-fetal-diagnosis-and-treatment/north-american-fetal-therapy-network-fetal-myelomeningocele-repair-registry, accessed on 19 September 2024) has addressed this gap by establishing the Fetal Myelomeningocele Consortium. This consortium aims to create a network of institutions performing fetal spina bifida repair and to develop a comprehensive prospective multicenter registry of maternal–fetal surgery cases for fetal spina bifida across all participating centers. The data collected through this registry will be invaluable in improving our understanding of long-term outcomes and in enabling clinical teams to offer families detailed information about their experiences with fetal spina bifida repair. We strongly encourage the inclusion of patients with obesity in this registry, as their data will contribute valuable insights that could enhance patient management and outcomes, ultimately supporting the expansion of access to this important intervention.

The exclusion of individuals with obesity from eligibility for fetal surgeries raises significant ethical concerns related to equity and access to medical care. Obesity-related risks, though serious, should not preclude fetal surgical interventions a priori. Instead, they should prompt personalized decision making and the integration of modern approaches to monitoring, prevention, and treatment. Many studies in the field of fetal surgery highlighted that with tailored technical adjustments, the disparities in surgical outcomes between patients with and without obesity can be minimized [18,19,20,22,23,24]. For example, in twin pregnancies complicated by twin-to-twin transfusion syndrome, studies have demonstrated that obesity does not significantly impact maternal or neonatal outcomes in cases of fetoscopic laser photocoagulation when appropriate adjustments, including modified patient positioning, lighter conscious sedation, longer introducers for fetoscopic procedures, and tailored anesthetic protocols, are made [22,23,24]. Although fetoscopic laser surgery and open fetal surgery for spina bifida differ substantially in their surgical approaches and technical requirements, this example illustrates how carefully implemented adjustments can help mitigate adverse outcomes for patients with obesity.

If a patient with obesity is considered for prenatal repair of fetal spina bifida, comprehensive counseling and transparent communication regarding the potential increased risks, including early delivery and obesity-related pregnancy complications, are imperative [17]. A multidisciplinary approach involving maternal–fetal specialists, surgeons, anesthesiologists, radiologists, and neonatologists is crucial to ensure comprehensive management. Detailed preoperative assessments, such as measuring abdominal wall thickness and carefully mapping the surgical site, can improve preparation and help anticipate potential challenges during surgery. Surgical adaptations may be required, including modified positioning, specific sedation protocols, and the use of equipment tailored to the needs of patients with higher BMIs [22]. The extended duration of both operative and anesthesia times in patients with obesity further highlights the importance of tailored anesthetic protocols and vigilant intraoperative monitoring [18,22]. For example, lighter conscious sedation might be necessary to enable patient cooperation, especially in situations where maternal breath holding is needed to manage upper airway obstruction, which can cause paradoxical abdominal wall motion during inspiration [22].

Given the higher incidence of fetal spina bifida in populations with obesity compared to the general population [6], this group requires more focused prenatal management. While obesity is an independent risk factor for perinatal mortality [25,26] and is associated with preterm birth [27], our results do not show an apparent higher risk compared to the MOMS trial. However, this finding should not diminish the importance of individualized care and risk assessment in managing these patients. As the prevalence of obesity continues to rise globally, ensuring equitable access to life-saving fetal interventions and optimizing outcomes for all patients, regardless of BMI, must remain a priority in maternal–fetal medicine.

## 5. Conclusions

The field of fetal surgery faces significant challenges in addressing the needs of patients with obesity, despite the rising prevalence of this condition and its well-documented impact on maternal and fetal health. Current evidence suggests that open fetal surgeries for fetal spina bifida in patients with obesity may be feasible, but the limited sample sizes underscore the need for more extensive research to validate these findings and ensure their generalizability.

Future research must focus on prospectively assessing the outcomes of prenatal repair of fetal spina bifida in patients with obesity using larger sample sizes across multiple major fetal centers. Additional outcomes should be investigated, including infection [28,29], thrombosis [28,30], and postpartum hemorrhage [28], as these complications are more prevalent in pregnancies with obesity. Future studies should aim to validate current findings, evaluate long-term outcomes, and establish clear guidelines for patient selection and management. Efforts must be directed toward refining surgical techniques to accommodate the anatomical differences in patients with obesity and developing tailored intervention protocols. By conducting rigorous prospective research with adequate statistical power, we can develop evidence-based protocols that address the unique challenges posed by maternal obesity in fetal surgery, ultimately improving care and outcomes for this growing patient population.

## Figures and Tables

**Table 1:** Characteristics of studies not adhering to body mass index-related MOMS criteria.

Reference	Study Institution(s)	Design	BMI ≥ 35 kg/m^2^, *n*	Outcomes	Main Results ^a^
Hilton et al., 2019 [17]	Children’s Hospital Colorado	Retrospective	11	Obstetrical outcomes: pPROM, pulmonary edema, hysterotomy dehiscence at delivery Fetal–neonatal outcomes: perinatal death, GA at birth, placement of shunt	Descriptive results (no comparison group). Mean GA at delivery in patients with BMIs of 35–40 kg/m^2^ was 2 weeks earlier compared to the one reported in the MOMS trial. No other adverse effects associated with a BMI ≥ 35 kg/m^2^ were reported.
Moldenhauer et al., 2020 [18]	Children’s Hospital of Philadelphia	Retrospective	14	Surgical outcomes: operative time, need for alloderm skin patch, intraoperative complications, maternal EBL, pulmonary edema, maternal LOS post-fetal surgery, wound complications Obstetrical outcomes: GDM, membrane separation, pPROM, spontaneous preterm labor, preterm delivery, GA at delivery, hysterotomy dehiscence at delivery, maternal EBL at delivery, wound complications at delivery, stillbirth Neonatal outcomes: birthweight, perinatal death, NICU LOS, shunt placement at 12 months	A BMI ≥ 35 kg/m^2^ was associated with a significant increase in operative time (*p* < 0.0001), GDM (*p* = 0.002), and a significant decrease in preterm delivery at <37 weeks. No significant differences in neonatal outcomes.
Moehrlen et al., 2023 [19]	Zurich Center for Fetal Diagnosis and Therapy	Retrospective	12 (2010–2020) ^b^	Shunt rate, GA at birth, lung embolism, seroma	No significant differences.
Zepf et al., 2024 [20]			18 (2010–2022)	Five levels of composite maternal and fetal outcomes	No significant differences.

^a^ Results from comparison with populations without obesity. ^b^ This cohort is likely already included in Zepf et al., 2024 [20]. MOMS (Management of Myelomeningocele Study); GA, gestational age; LOS, length of stay; EBL, estimated blood loss; GDM, gestational diabetes mellitus; pPROM, premature prelabor rupture of membranes; NICU, neonatal intensive care unit; SD, standard deviation; BMI, body mass index.

**Table 2:** Pooled proportions and means for reported obstetrical and perinatal outcomes in fetal spina bifida repair with BMI ≥ 35 kg/m^2^ along with MOMS Trial data for patients with BMI < 35 kg/m^2^.

Outcome	^a^ BMI ≥ 35 kg/m^2^			MOMS Trial
	Studies (*n*)	PP or PM 95% CI	I^2^ (%)	Incidence (%) or Mean ± SD [Reference]
Duration of surgery (minutes)	2	118.35 (58.67–178.03)	0.99	104.85 ± 24.45 [21]
Membrane separation (%)	2	10.32 (3.34–27.69)	0	26 [10]
pPROM (%)	2	28.00 (13.98–48.22)	0	28 [21]
Preterm delivery < 37 GWs (%)	2	81.95 (29.15–98.04)	0.63	79 [10]
Maternal pulmonary edema (%)	3	3.32 (0.67–14.87)	0	6 [10]
Perinatal mortality (%)	3	6.08 (1.76–18.92)	0	3 [10]
Focal or complete dehiscence (%)	3	3.32 (0.67–14.87)	0	1 [10]
GA at birth (weeks)	3	34.31 (32.28–36.33)	0.78	34 ± 3 [21]
Birth weight (grams)	2	2651.5 (2473.67–2829.35)	0	2383 ± 688 [10]
Need for shunt (%)	2	40.09 (23.05–59.93)	0	40 [10]

^a^ Results of meta-analysis of studies with patients having BMI ≥ 35 kg/m^2^ (Table 1). BMI, body mass index; PP, pooled proportion; PM, pooled mean; pPROM, premature prelabor rupture of membranes; GWs, gestational weeks; GA, gestational age; SD, standard deviation.
